# 

**Published:** 1868

**Authors:** 


Vol. IX A. JULY & AUGUST, 1868. No. 3.

### ATLANTA

### Medical and Surgical

### JOURNAL.

NEW SERIES.

S. G. O. INDEXED

EDITED BY

**J. G. WESTMORELAND, M. D.,**
*Professor of Materia Medica and Therapeutics in the Atlanta Medical College.*

**W. F. WESTMORELAND, M. D.,**
*Professor of the Principles and Practice of Surgery in the Atlanta Medical College.*

AND

**J. M. JOHNSON, M. D.**

*Professor of Physiology in the Atlanta Medical College.*

**Pax et scientia, sed veritas sine timore.**

**PUBLISHED BIMONTHLY AT $3 PER ANNUM, IN ADVANCE**

ATLANTA, GA.:

PRINTED AT THE ATLANTA INTELLIGENCER BOOK & JOB OFFICE.

1868.

Postage 36 cts. a year, pre-paid quarterly. Postmasters are requested to comply with the law, by giving us notice when the Journal is refused at their Offices.

